# Local Binary Patterns Descriptor Based on Sparse Curvelet Coefficients for False-Positive Reduction in Mammograms

**DOI:** 10.1155/2018/5940436

**Published:** 2018-09-25

**Authors:** Meenakshi M. Pawar, Sanjay N. Talbar, Akshay Dudhane

**Affiliations:** ^1^Department of Electronics and Telecommunication, SVERI's College of Engineering, Pandharpur, Solapur, Maharashtra, India; ^2^Department of Electronics and Telecommunication Engg., S.G.G.S.I.E. & T, Nanded, Maharashtra, India

## Abstract

Breast Cancer is the most prevalent cancer among women across the globe. Automatic detection of breast cancer using Computer Aided Diagnosis (CAD) system suffers from false positives (FPs). Thus, reduction of FP is one of the challenging tasks to improve the performance of the diagnosis systems. In the present work, new FP reduction technique has been proposed for breast cancer diagnosis. It is based on appropriate integration of preprocessing, Self-organizing map (SOM) clustering, region of interest (ROI) extraction, and FP reduction. In preprocessing, contrast enhancement of mammograms has been achieved using Local Entropy Maximization algorithm. The unsupervised SOM clusters an image into number of segments to identify the cancerous region and extracts tumor regions (i.e., ROIs). However, it also detects some FPs which affects the efficiency of the algorithm. Therefore, to reduce the FPs, the output of the SOM is given to the FP reduction step which is aimed to classify the extracted ROIs into normal and abnormal class. FP reduction consists of feature mining from the ROIs using proposed local sparse curvelet coefficients followed by classification using artificial neural network (ANN). The performance of proposed algorithm has been validated using the local datasets as TMCH (Tata Memorial Cancer Hospital) and publicly available MIAS (Suckling et al., 1994) and DDSM (Heath et al., 2000) database. The proposed technique results in reduction of FPs from 0.85 to 0.02 FP/image for MIAS, 4.81 to 0.16 FP/image for DDSM, and 2.32 to 0.05 FP/image for TMCH reflecting huge improvement in classification of mammograms.

## 1. Introduction

Breast cancer is the most common cancer disease among women across worldwide. It is the leading cause of deaths for women suffering from cancer disease in India. It is estimated that breast cancer cases in India would reach to as high as 1,797,900 by 2020 [1]. Rising rate of incidences can cause high mortality. This is due to lack of awareness about breast screening, late reporting, and insufficient medical access [2]. This fact brings a concern and necessity that screening for breast cancer is prudent in its early stage to confirm longer survival. Among all techniques, namely, mammography, tomosynthesis, ultrasonography, computed tomography, and magnetic resonance, mammography is the most reliable and accepted modality by radiologist for preliminary examination of breast cancer due to cost benefits and accessibility [3–5]. The diagnosis of breast cancer using mammogram by radiologist varies from expert to expert as symptoms are misinterpreted or overlooked, due to the tedious task of screening mammograms. Study reveals that 10% to 30% of the visible cancers on mammograms are overlooked, and only 20% to 30% of biopsies are positive [6–8]. Biopsies are traumatic in nature and costly; therefore, computer aided detection and diagnosis (CAD) systems combined with expert radiologists' experience would provide more comprehensive diagnosis [9]. Detailed survey about the research in the design of CAD systems has been given in next section.

## 2. Literature Survey

The design and development of CAD system is an important progressive area of research for contrast enhancement for better visualization and clarification [10–12], pectoral muscle removal, segmentation for better delineation of region of interest (ROI), extraction of features, and classification [13, 14]. The segmentation method is classified as region based, contour-based, and clustering method [15]. The region and contour-based methods are popularly used by many researchers. Görgel et al. [16] developed Local Seed Region Growing-Spherical Wavelet Transform (LSRG–SWT) algorithm using local dataset and MIAS [17] with classification accuracy of 94% and 91.67%, respectively. Pereira et al. [18] presented segmentation and detection of masses in mammogram using wavelet transform and genetic algorithm that provides FP rate of 1.35 FP/image and sensitivity of 95% using DDSM [19]. Rouhi et al. [20] studied segmentation using region growing, Cellular Neural Network (CNN), and ANN. The result of classification varied from 80 to 96%, which is the main weakness of their study. Berber et al. [21] proposed Breast Mass Contour Segmentation (BMCS) approach and showed 6 FPR for local dataset. Hybrid level set segmentation method [22] based on combination of region growing and level set was used to segment tumor. The results showed that the sensitivity varied from 78 to 100% due to the presence of artifact in the MIAS database. The difficulties in region and contour-based segmentation methods are the appropriate initialization of seed point and contour position.

Several researchers have implemented clustering method like K-means and Fuzzy C-means (FCM) for breast abnormality segmentation [3, 23]. However, they have limitations in terms of learning abilities. Learning-based techniques such as Self-organizing map (SOM) [24] have been successfully used in medical image segmentation [25]. The success of SOM in medical image segmentation has inspired the researcher to choose it for mammogram segmentation. Many of the times the tumor-segmented regions are not the abnormal tissues (cancerous region), and they are known as false positives (FPs). This FP consumes much time of radiologists and results into unnecessary biopsies. Thus, reducing the FPs is an open research problem and various researchers have proposed FP reduction algorithms to improve the specificity of the CAD systems [5, 9, 23, 26–31]. Usually, FP reduction algorithm is postprocessing step of a CAD system with two stages namely: Feature extraction and Classification. Various methods have been developed for feature extraction based on wavelets [8, 18, 32], curvelet [33, 34], Gabor [35, 36], morphological descriptors [20], textural analysis [26, 27, 30, 32], histogram [4, 5, 7, 29, 37–40], etc. The segmentation error can reduce the performances of morphological descriptor. When Gray Level Co-occurrence Matrix (GLCM) from normal and abnormal region in dense mammogram is same, texture descriptor overlaps that leads to more number of FPs [37]. Ojala et al. proposed local binary patterns (LBPs) [41] for textural feature extraction which works well in feature extraction as compared to morphological descriptor and GLCM-based textural descriptor. LBP descriptor can be considered as local microstructures, namely, edges, flat areas, spots, etc. Variants of LBP have been proposed by various researchers to achieve rotation and intensity invariant features. Also, LBP is computationally efficient and extracts robust features; therefore, LBP descriptors have been widely applied in FP reduction and classification methods for mammogram images [29, 37, 39, 40]. However, LBP descriptor does not provide the directional information of local micropattern. Therefore, transform technique such as curvelet combined with LBP was used to extract features. Various curvelet-based approaches have been proposed in the literature [8, 33, 34, 42] which conclude that curvelet outperforms as compared to wavelet transform.

In this work, novel method of extracting sparse curvelet subband coefficients by incorporating the knowledge of irregular shape of masses as they appear in sparse matrix and calculating LBP features has been presented. Therefore, this paper presents scheme as follows:

1. Preprocessing of mammogram image for contrast enhancement using local entropy maximization-based image fusion algorithm and removal of background noise
2. Cluster-based segmentation of mammograms using SOM and extract tumor regions, i.e., ROI)
3. FP reduction: extraction of sparse curvelet subband coefficients and computation of LBP descriptor to classify true positives and false positives to improve performance of CAD system using MIAS [17], DDSM [19], and Tata Memorial Cancer Hospital (TMCH) datasets.

The organization of paper is as follows: Sections 1 and 2 illustrate the introduction and literature review on automatic segmentation and extraction of abnormal masses (i.e., tumor region) as well as FP reduction methods.  Section 3 presents the proposed methodology for SOM based segmentation of mammograms followed by novel false positive reduction in detail.  Section 4 depicts the experimental results and discussions on three benchmark datasets. Finally, Section 5 concludes the proposed approach for accurate extraction of abnormal masses (i.e., tumor region) by excluding the FPs.

## 3. Methodology

**Figure 1:**
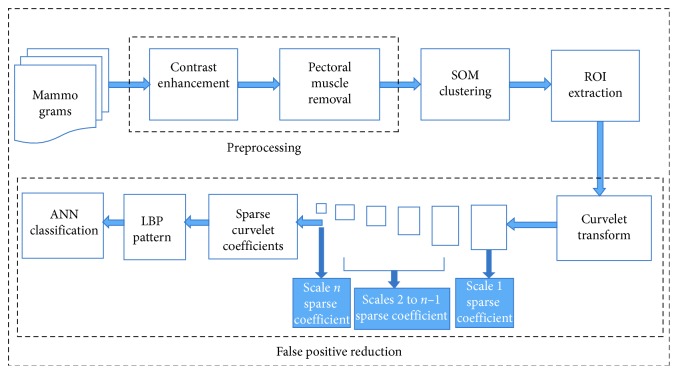
Schematic architecture for automatic breast cancer detection.

The block schematic of proposed integrated method for automatic detection of breast cancer using sparse curvelet coefficient-based LBP descriptor has been shown in Figure 1.

### 3.1. Preprocessing

**Figure 2:**
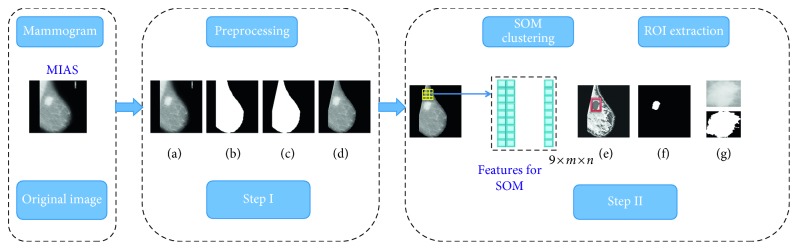
Steps for mammogram processing (a) enhanced mammogram, (b) binary mask, (c) pectoral removal, (d) pectoral removed mammogram, (e) clustered image, (f) cluster of interest, and (g) ROI extraction.

The mammogram images are low-dose x-ray images so they have poor contrast and suffer from noises. The preprocessed mammogram image as shown in Figures 2–2 represents preprocessing of mammogram, and Figures 2–2 represents SOM clustering and ROI extraction.

#### 3.1.1. Local Entropy Maximization-Based Image Fusion: Contrast Enhancement

**Figure 3:**
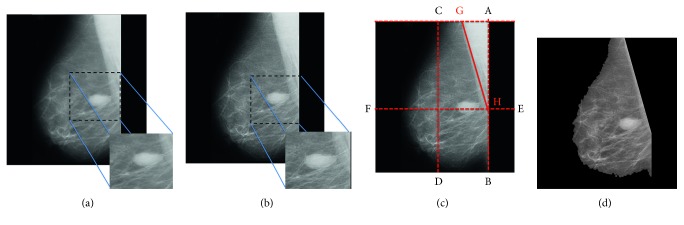
Preprocessing. (a) Original image from MIAS database. (b) Contrast-enhanced mammogram using local entropy maximization. (c) Process of pectoral muscle removal. (d) Pectoral muscle removed mammogram.

The contrast enhancement of the mammogram is performed using local entropy maximization [12] for better segmentation. Here, original image is given to the contrast limited adaptive histogram equalization (CLAHE) algorithm to get the second input to our image fusion algorithm. Further, original image along with the CLAHE has been given to the image fusion algorithm. Procedure of the image fusion has been given in Algorithm 1. We have used local entropy as a fusion rule given by the following equation:

(1) $$\begin{array}{c} \text{ENT}=-\overset{255}{\underset{k=0}{\sum }}p\left(k\right)\log\left(p\left(k\right)\right), \end{array}$$

where ENT is the local entropy and *p*_org(*k*) and *p*_CLAHE(*k*) are the probability of *k*^th^ pixel from 5 × 5 sliding window [12]. Here, both high frequency components from original mammogram and CLAHE mammogram have been fused using maximum entropy criteria.  Figure 3(b) presents contrast-enhanced mammogram using local entropy maximization-based image fusion.

#### 3.1.2. Pectoral Muscle Removal

Pectoral muscle suppression has been performed by defining rectangle as suggested in [14] (Figure 3(c)). It illustrates the rectangle (ABDC) and fixes the points G and has intensity variation and joins them for pectoral muscle suppression.  Figure 3(d) illustrates pectoral muscle removed image to avoid discrepancies in the algorithm because of similar intensities present between pectoral muscle and masses.

### 3.2. SOM Clustering

SOM is a special type of neural network designed to map the input image of size *N*_*x*_ × *N*_*y*_ to *M* clusters based on their characteristic features [25]. For SOM, the image (I) is converted into a feature vector *f*={*f*_1_, *f*_2_,…, *f*_*m*_}, where *m* is the number of features. In this experiment, we have trained SOM with *M* = 4 clusters using *p*=9 neighbourhood features such as given a centre pixel (*g*_c_) in the image, the neighbourhood features are computed as given in the following equation:

(2) $$\begin{array}{c} F\left(1,p\right)=g_{p},\;p\in \left[1,n\right], \end{array}$$

where *n* is the number of neighbourhood (3 × 3 window), *g*_*p*_ is the neighbourhoods, and *F* is the feature vector corresponds to centre pixel *g*_c_. The selection of 3 × 3 window pixel is based on [43] to capture local details.

At the start, weight vector *W*_i_={*w*_i1_, *w*_i2_,…, *w*_i*m*−1_} is random and updated as the network learns. The minimum Euclidean distance ‖*f* − *W*_i_‖ is described as the best matching component or winner node ‖*f* − *W*_c_‖ and described as

(3) $$\begin{array}{c} \left\|f-W_{\text{c}}\right\|=\min_{i}\left\{\left\|f-W_{\text{i}}\right\|\right\}. \end{array}$$

Weight vector for winning output neuron and its neighboring neurons are updated as

(4) $$\begin{array}{c} W_{\text{i}}\left(t+1\right)=W_{\text{i}}\left(t\right)+N_{\text{ci}}\left(t\right)\left(f\left(t\right)-W_{\text{i}}\left(t\right)\right), \end{array}$$

where *t*=1, 2,… is time coordinate. The function *N*_ci_(*t*) is the neighbourhood kernel function and expressed as

(5) $$\begin{array}{c} N_{\text{ci}}\left(t\right)=\eta \left(t\right)\exp\;\left(-\frac{\left\|m_{\text{c}}-m_{\text{i}}^{2}\right\|}{2\sigma^{2}\left(t\right)}\right), \end{array}$$

where *η*(*t*) is the learning rate, *σ*(*t*) is a width of kernel that corresponds to neighbourhood neurons around node c and *m*_c_ and *m*_i_ corresponds to location vectors of nodes c and i.

**Figure 4:**
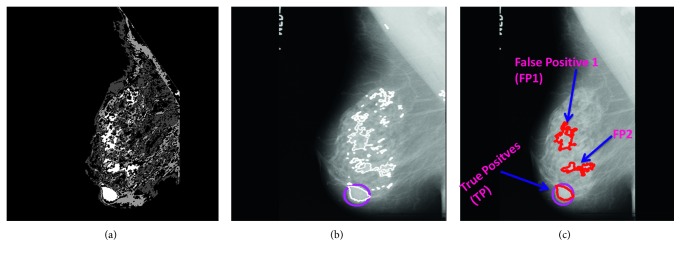
FP reduction by thresholding (a) clustered image, (b) clusters boundaries marked on original image, and (c) clusters after thresholding.

Figures 4(a) and 4(b) represent cluster map and cluster boundaries marked on mammogram. After the several observations for known areas, it was empirically noticed that number of pixels of range or pixel level threshold (PLT based on pixel count in TP) as 450 to 31,500; 16,000 to 2,00,000; and 4,000 to 2,00,000 consist of abnormality for MIAS, DDSM, and TMCH database, respectively, which is verified from the expert. The size of the tumor is varying because of the mammogram size of 1024 × 1024 pixels for MIAS, 2728 × 3920 pixels to 4608 × 6048 pixels for DDSM, and 2294 × 1914 or 4096 × 3328 pixels for TMCH datasets. Therefore, cluster regions below or above the specified threshold are discarded and the remaining region is marked as true positive (TP) as shown in Figure 4.  Figure 4(a) shows the clustered image using SOM; Figure 4(b) shows the cluster boundaries marked on original image.

We can see that there are many FPs along with TP (marked by pink color) which are reduced using pixel level threshold (PLT based on pixel count in TP) as explained above.  Figure 4(c) shows the filtered result using PLT.

### 3.3. ROI Extraction

**Figure 5:**
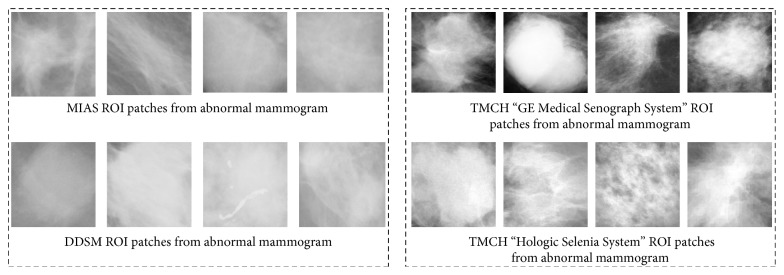
Variable sizes ROIs from MIAS, DDSM, and TMCH datasets.

After SOM clustering (initial segmentation), the next step is to classify the detected regions into TP and FP by using proposed local sparse curvelet features (LSCF) followed by ANN classifier. To do so, initially, we have extracted ROIs from detected regions by SOM clustering and manually categorized into TP and FP. We collected these ROIs from three different datasets according to their maximum height and maximum width using connected components e.g., region marked in Figure 4(c). Therefore, their patch size is different as shown in Figure 5, ROIs for MIAS, DDSM, and TMCH dataset. Further, these extracted patches have been used to train the ANN for the task of FP reduction.

### 3.4. False-Positive (FP) Reduction

After ROI extraction, FP reduction algorithm performs computation of proposed local sparse curvelet features (LSCF) followed by ANN classifier.

#### 3.4.1. Proposed Algorithm

LBP [43] was proposed as LBP descriptor computation at circular neighbourhood which is called as uniform LBP (ULBP) descriptor and expressed as

(6) $$\begin{array}{c} {\text{ULBP}}_{\left(P,R\right)}=\left\{\begin{array}{cc} \overset{P-1}{\underset{n=1}{\sum }}S & \text{if }U\left(LBP_{\left(P,R\right)}\right)\leq 2,\\ P+1 & \text{otherwise}, \end{array}\right. \end{array}$$

where

(7) $$\begin{array}{c} U\left({\text{LBP}}_{\left(P,R\right)}\right)=\left|S\left(g_{P-1}-g_{\text{C}}\right)-S\left(g_{0}-g_{\text{C}}\right)\right|+\overset{P-1}{\underset{n=1}{\sum }}\left|S\left(g_{P}-g_{\text{C}}\right)-S\left(g_{P-1}-g_{\text{C}}\right)\right|. \end{array}$$

**Figure 6:**
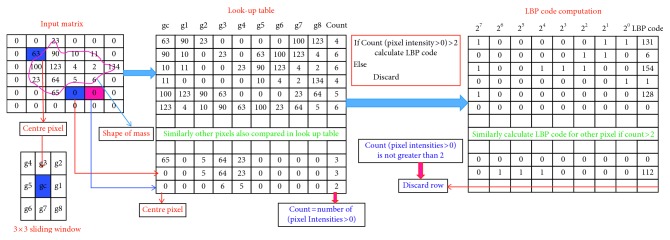
Lookup table approach for LBP computation from shape of mass in ROI.

**Figure 7:**
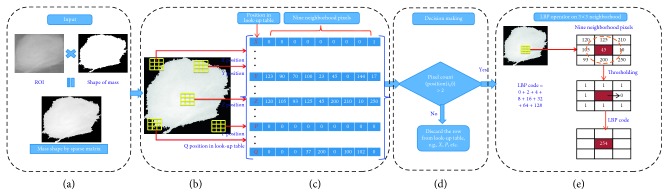
Process for computation of LBP descriptor from shape of mass in ROI. (a) Original image, (b) 3 × 3 window for selection of foreground pixels, (c) lookup table, (d) decision making process, (e) LBP computation from selected foreground pixels.

Computation of LBP based on actual shape of mass according to sparse matrix has been shown in Figure 6, where it takes pixels related to shape of mass which are called as foreground pixels and rejects the other pixels called as background pixels. The proposed algorithm uses foreground pixels only for LBP computation, and this will tend to number of pixel reduction in LBP computations. Therefore, identification of foreground and background pixels is an important step which is performed using lookup table approach. The identification of foreground and background pixel is based on number of nonzero pixels in the lookup table, i.e., if count of sliding window nonzero pixels is greater than 2, count(*p*(*i*, *j*)) > 2 is identified as foreground and LBP is estimated. On the other hand, if count of sliding window nonzero pixels is less than 2, count(*p*(*i*, *j*)) < 2 is identified as background and LBP would not be estimated and rejected from lookup table. Nonzero pixels provide actual shape of mass and are taken for LBP computations. Graphical representation of proposed algorithm for LBP descriptor computation using foreground pixels has been given in Figure 7 and the algorithm has been described in Algorithm 2.

#### 3.4.2. The Fast Discrete Curvelet Transform (FDCT)

The authors [44] have introduced computationally simple and efficient Fast Discrete Curvelet Transform (FDCT). We have preferred wrapping-based FDCT approach in proposed work, as it is faster. The curvelet coefficients *C*^D^(*j*, *l*, *k*) represented by scale *j*, angle *l*, and spatial location *k* can be written as

(8) $$\begin{array}{c} C^{\text{D}}\left(j,l,k_{1},k_{2}\right)=\overset{n_{1}=N_{1}}{\underset{n_{1}=1}{\sum }}\overset{n_{2}=N_{2}}{\underset{n_{2}=1}{\sum }}I\left[n_{1},n_{2}\right]\varphi_{j,l,k_{1},k_{2}}^{\text{D}}\left[n_{1},n_{2}\right]. \end{array}$$

**Figure 8:**
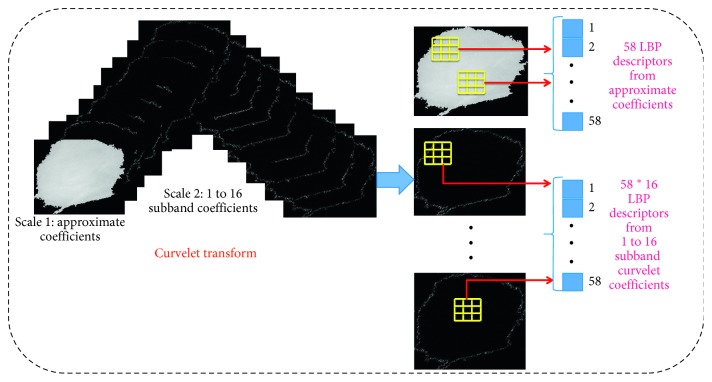
LBP code computation using sparse curvelet subband coefficients.

Figure 8 illustrates LBP code computation based on sparse curvelet coefficients; ROI decomposes using curvelet transform with scale orientations *l* of 16° and scale of 2 as the database consists of minimum ROI size of 25 × 22 pixels. Curvelet transform with scale orientations *l* of 16° and scale of 2 produces 1+16=17 different subbands based on subband division. Further, each curvelet subband coefficients have been represented using lookup table using 3 × 3 sliding window, and if the row in the lookup table identifies foreground coefficient, then LBP is computed with radius *R* = 1 and *P* = 8 neighboring pixels as shown in Algorithm 2; total 58 LBP features have been obtained from foreground curvelet subband coefficients. Therefore, total 986 LBP features have been extracted from 17 curvelet subbands. It can be observed from Figure 8, curvelet subbands also provide shape of mass in 16 different directions so that the directional information can be associated with LBP features. Kanadam et al. [3] used concept of sparse ROI; similarly, we have extended it for sparse curvelet subband and LBP features computation.

### 3.5. Classification

**Figure 9:**
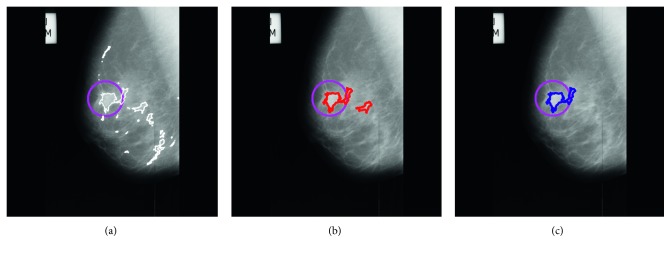
(a) FP reduction by clusters marked on original image, (b) FP reduction by thresholding, (c) FP reduction by sparse curvelet coefficient-based LBP, and ANN.

In this work, we have analyzed extracted ROI from mammogram using normal-abnormal, benign-malignant, and normal-malignant classes with ANN, SVM, and KNN classifiers. The detailed description of ANN classifier has been given in [45, 46]. To evaluate performance of the proposed system, we have used 3-fold cross validation where database is randomly divided into three sets and accuracy is calculated for each set. The final accuracy of the system is average of accuracy of each of three sets. However, it will not be fair to compare 3-fold cross validation result of SVM and KNN classifier with ANN, because ANN classifier is tested on only one set of images (33% for training, 33% for testing, and 33% for validation). Thus, to do fair comparison, we have trained ANN using input layer (986 neuron) over three different sets (which are considered in SVM and KNN) and calculated its average accuracy. Our proposed false positive reduction algorithm illustrates in Figures 9(a)–9(c).  Algorithm 3 summarizes flow of the proposed method for FP reduction in mammograms.

## 4. Experimental Results and Discussions

The proposed method has been tested and validated using three classifiers and three clinical mammographic image datasets.

### 4.1. Data Sets

#### 4.1.1. Mammographic Image Analysis Society (MIAS) Database

The mini-MIAS [17] database consists of 322 mammograms, each having 1024 × 1024 pixels and annotated like background tissue character, class, severity, center of abnormality, and radius of circle for abnormality. This database includes 64 benign, 51 malignant, and 207 normal cases, which have been taken for experimentation.

#### 4.1.2. Digital Database for Screening Mammography (DDSM)

The DDSM [19] dataset consists of 2500 studies and is composed of cranial-caudal (CC) and mediolateral-oblique (MLO) views of mammographic image for left and right breast, annotated with ACR breast density, type of abnormality, and ground truth. Randomly selected 150 abnormal and 100 normal cases from both HOWTEK and LUMISYS scanner of 12 bits per pixel resolution have been subjected for experimentation.

#### 4.1.3. The Tata Memorial Cancer Hospital (TMCH)

This dataset [47] contains 360 full-field digital mammograms (FFDMs) comprising 180 CC views and 180 MLO views from right and left breast acquired from 90 randomly selected patients. It is composed of 180 verified malignant and 180 normal breast images. It uses biopsy proven breast cancer patients' pathological data approved by the Institutional Research Ethics Committee of Tata Memorial Centre Hospital (TMCH), Mumbai, India. The ground truth marking on each abnormal mammogram is performed manually using the Histopathological Reports (HPR) of the respective patients and expert radiologist from TMCH, Mumbai. Approximately 35 patients are examined using “Hologic Selenia System” (Scanner1) gives 16-bit.

The remaining 55 patients were examined with “GE Medical Senograph System” (Scanner2) providing 8-bit true color mammogram image in DICOM format of 4096 × 3328 or 2294 × 1914 pixels each measuring size 50 × 50 *μ*m^2^.

### 4.2. Segmentation Evaluation and ROI Extraction

The segmentation using SOM that detects suspicious mass regions is considered as TP whereas from nonmass is taken as FP. From Table 1, it is clear that total suspicious ROI (including TP & FP) of 381 for MIAS, 1343 for DDSM, and 1009 for TMCH have been taken for evaluation our proposed algorithm for FP reduction.

From extracted ROIs, the minimum patch size is 25 × 22 pixels whereas the maximum size is 1152 × 1356 pixels. Tables 2 and 3 represent curvelet subband coefficients from 17 subbands, and reduced coefficients based on lookup table approach are used to calculate LBP features. It has been observed during experimentation that the curvelet coefficients on an average are reduced for sparse LBP by 14%, 32%, 33%, and 34% for MIAS, DDSM, TMCH: Scanner1, and TMCH: Scanner2, respectively. It may be noticed that reduction in curvelet coefficients for every ROI is not fixed. It completely depends upon the shape of the ROI as per the sparse matrix. Tables 2 and 3 do not represent exact reduction in pixels for complete database, but they exhibit pixel reduction for sample mammograms.

### 4.3. Classifier Evaluation and False-Positive Reduction

**Figure 10:**
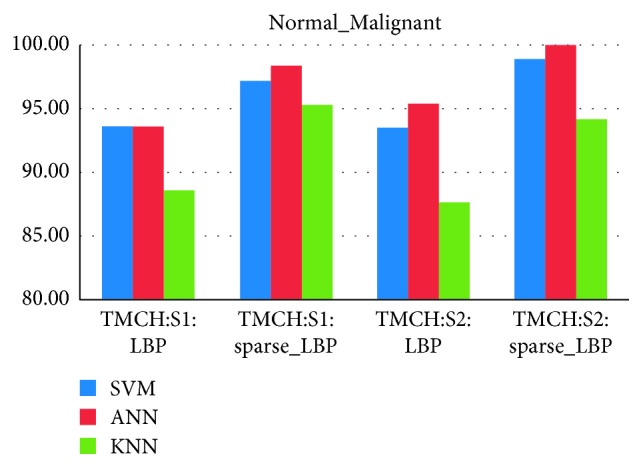
Average classification rate for TMCH dataset.

**Figure 11:**
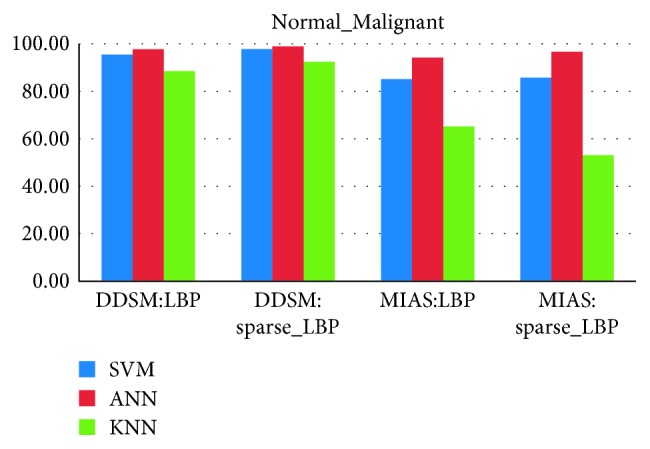
Average classification rate for MIAS and DDSM dataset.

**Figure 12:**
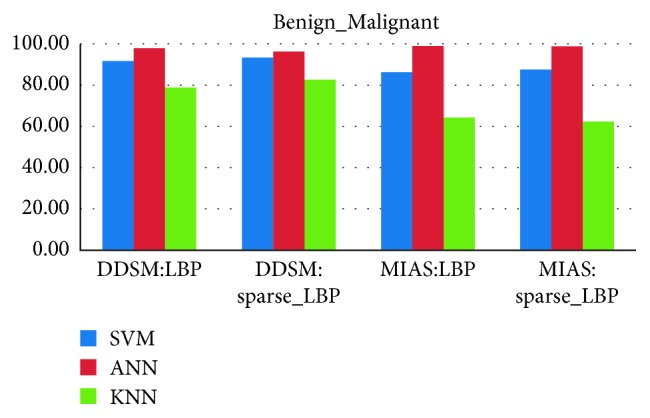
Average classification rate for MIAS and DDSM dataset.

**Figure 13:**
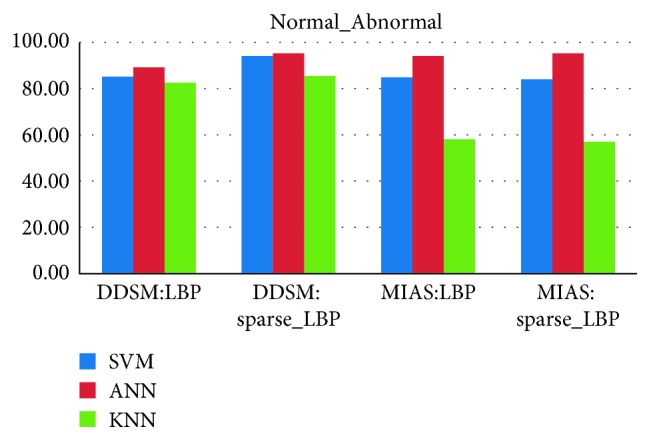
Average classification rate for MIAS and DDSM dataset.

From Figures 10–13, the best classification accuracy of 98.57 % has been obtained for MIAS in benign versus malignant classification, whereas 98.70% for DDSM, 98.30% for TMCH: Scanner1, and 100% for TMCH: Scanner2 classification accuracies have been obtained in normal versus malignant classification. The classification performance of ANN has improved from 6% to 43% for different databases as compared to KNN classifier, whereas there is little improvement about 7% compared with SVM classifier. The performances of both proposed sparse LBP and LBP computation on curvelet subbands are nearly same; therefore, the proposed algorithm can be efficiently implemented in CAD system with lesser number of curvelet coefficients.

Data augmentation has been used for some classes to maintain balance between two classes, to improve performance, and to learn more powerful model.  Table 4 explains the FP reduction with the use of curvelet-based LBP features and ANN. It has been observed that FP reduced from 0.85 to 0.02 FP/image in MIAS, 4.81 to 0.02 FP/image in DDSM and 2.32 to 0.13 FP/image in TMCH.

Similarly, Table 5 shows the reduction in FPs as 0.85 to 0.01 FP/image for MIAS, 4.81 to 0.03 FP/image for DDSM, and 2.32 to 0.00 FP/image for TMCH using sparse curvelet coefficient-based LBP features. The results show the effectiveness of sparse curvelet coefficient-based LBP and ANN. From Table 6, the best value of AUC = 0.99 is obtained in benign versus malignant classification for MIAS, AUC = 0.98 in benign versus malignant in case of DDSM, AUC = 0.94 in normal versus malignant in case of TMCH: Scanner1, and AUC = 0.96 in normal versus malignant classification in TMCH: Scanner2 using ANN and curvelet subband-based LBP features. The worst performance of AUC = 0.53 for MIAS is obtained with the proposed algorithm using KNN classifier as shown in Table 7. Similarly, from Table 7, the best value of AUC = 0.98 is obtained in TMCH: Scanner1, AUC = 1 is obtained in TMCH: Scanner2 database for normal versus malignant classification, AUC = 0.98 in benign versus malignant classification is attained in MIAS database, and AUC = 0.98 is achieved for normal versus malignant classification in DDSM database using ANN classifier for sparse curvelet subband-based LBP features.

**Figure 14:**
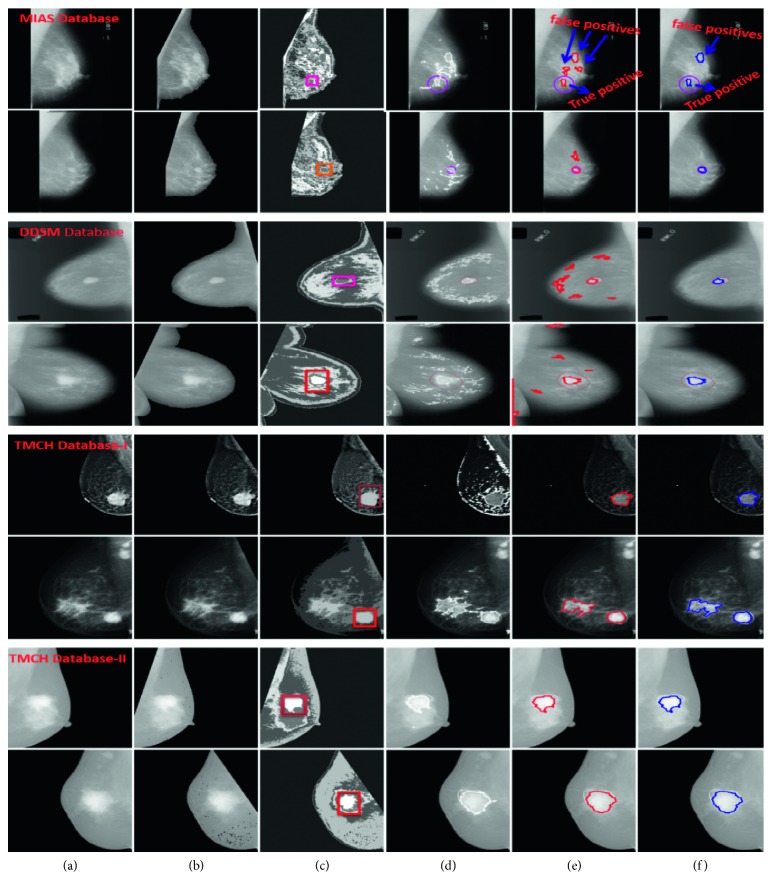
Representation of fully automatic CAD system for breast cancer using (a) sample mammograms from MIAS, DDSM, and TMCH datasets, (b) preprocessed mammograms, (c) clustered image, (d) TP and FP marked on mammogram, (e) TP marked by thresholding, (f) TP marked by using LBP descriptor based on sparse curvelet coefficients.

However, from Table 7, it should be noted that the performance of proposed algorithm is the best using ANN classifier.  Figure 14 represents automated CAD system for breast cancer diagnosis with sample mammograms.

Table 8 provides comparative study of methods developed for breast tissue classification. The proposed method provides best results in terms of AUC and reduction of number of FPs as 0.85 to 0.01 FP/image for MIAS, 4.81 to 0.03 FP/image for DDSM, and 2.32 to 0.00 FP/image for TMCH. The earlier reported work uses the fixed patch size-based approach which limits the automatic CAD system scope whereas proposed system provides complete solution to CAD system right from automatic tumor patch segmentation to reduction in FPs and final representation of mammogram with TP marked on it. It will drastically reduce the radiologist work by location tumor directly on mammogram.

## 5. Conclusion

A fully automatic CAD system, which can accurately locate the tumor on a mammogram and reduces FPs, has been proposed. The developed CAD system consists of preprocessing, SOM clustering, ROI extraction, sparse LBP feature computation based on sparse Curvelet coefficients, and finally, FP reduction using ANN classifier.

The proposed algorithm presents a novel concept of extraction of curvelet coefficients according to irregular shape of mass is called as sparse curvelet coefficients and computation of LBP. The analysis proves that the FPs are reduced significantly from 0.85 to 0.01 FP/image for MIAS, 4.81 to 0.03 FP/image for DDSM and 2.32 to 0.00 FP/image for TMCH. The ANN classifier showed best results as AUC = 0.98 and accuracy = 98.57% for MIAS in benign-malignant classification, AUC = 0.98 and accuracy = 98.70% for DDSM in normal-malignant classification, AUC = 0.98 and accuracy = 98.30% for TMCH: Scanner1, and AUC = 1 and accuracy = 100% for TMCH: Scanner2 in normal-malignant classification as compared with SVM and KNN classifier. The performance of LBP features and LBP features based on sparse curvelet coefficients are nearly same which show that the proposed algorithm is suitable for cancer breast tissue diagnosis.

In future, the reduced curvelet coefficients can be used to extract local ternary patterns and other local descriptor and local directional patterns, etc. The present work deals with mammogram with single mass; this can be further extended for multiple mass models with multiple LBP features based on sparse curvelet coefficients.

## Figures and Tables

**Algorithm 1:**
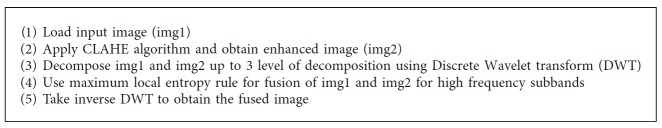
Image fusion for contrast enhancement.

**Algorithm 2:**
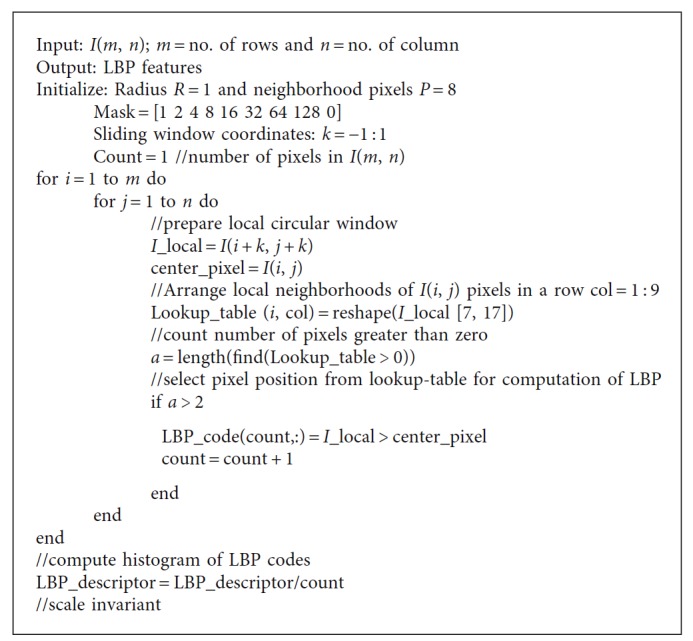
Algorithm for LBP feature computation based on shape of mass in ROI as.

**Algorithm 3:**
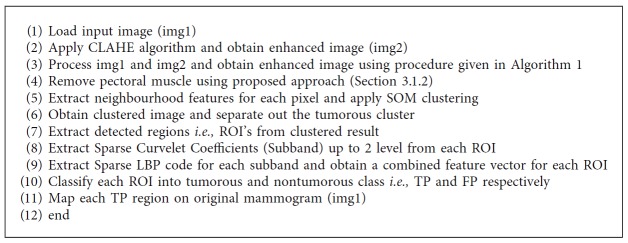
Summary of proposed method for FP reduction in mammograms.

**Table 1:** Result of SOM segmentation.

Dataset used	Result of SOM clustering and threshold				TPR (true-positive rate) = TP/#lesions	FPPI (false-positive per image) = FP/#images
	Mass		Segmented nonmass (FP)	Total (#) images		
	Segmented (TP)	Lost				
MIAS	108	7	273	322	(108/115) = 0.94	(273/322) = 0.85
DDSM	140	10	1203	250	(140/150) = 0.93	(1203/250) = 4.81
TMCH	172	8	837	360	(172/180) = 0.95	(837/360) = 2.32

**Table 2:** Reduction in curvelet coefficients for sample mammograms from MIAS and DDSM dataset.

Sr. No.	MIAS				DDSM			
	ROI Size	Total number of curvelet coefficients from subbands	Total number of selected curvelet coefficients from subbands	% reduction in curvelet coefficients	ROI Size	Total number of curvelet coefficients from subbands	Total number of selected curvelet coefficients from subbands	% reduction in curvelet coefficients
1	124 × 138	1,03,911	77,133	25.77	192 × 187	2,16,729	1,68,333	22.33
2	179 × 138	1,50,123	1,26,142	15.97	294 × 291	5,18,267	3,66,680	29.25
3	51 × 116	36,815	33,421	9.22	145 × 207	1,81,663	1,48,765	18.11
4	83 × 83	42,449	36,653	13.65	169 × 168	1,71,873	1,54,752	9.96
5	84 × 76	39,115	35,815	8.44	182 × 248	2,72,517	2,19,822	19.34
6	74 × 83	37,767	34,448	8.79	213 × 349	4,49,783	3,01,359	33.00
7	53 × 64	20,969	18,621	11.20	578 × 412	14,33,195	6,62,072	53.80
8	70 × 44	18,899	16,610	12.11	215 × 219	2,86,429	2,42,935	15.18
9	80 × 66	32,409	29,454	9.12	420 × 428	10,82,461	5,01,209	53.70
10	69 × 86	36,783	33,552	8.78	203 × 307	3,75,871	2,66,829	29.01
11	59 × 116	42,019	38,442	8.51	226 × 262	3,57,209	2,76,763	22.52
12	81 × 101	50,637	46,122	8.92	159 × 194	1,87,563	1,48,741	20.70
13	41 × 84	21,427	18,907	11.76	718 × 686	29,61,127	7,62,561	74.25
14	69 × 141	60,641	52,427	13.54	409 × 550	13,52,439	9,04,829	33.10
15	60 × 62	22,925	20,475	10.69	524 × 375	11,84,671	7,66,522	35.30
16	96 × 101	59,647	54,235	9.07	311 × 275	5,17,433	3,97,737	23.13
17	136 × 139	1,13,727	66,352	41.56	319 × 320	6,14,129	4,12,606	32.81
18	55 × 94	31,359	28,305	9.74	313 × 447	8,42,855	5,10,482	39.43
19	157 × 140	1,32,373	1,07,000	19.17	291 × 517	9,07,903	6,37,412	29.79
20	156 × 130	1,23,007	90,834	26.15	370 × 837	18,64,889	8,98,236	51.83
Average		58,850	48,247	14	Average	7,88,950	4,37,432	32

**Table 3:** Reduction in curvelet coefficients for sample mammograms from TMCH Scanner1 and Scanner2 dataset.

Sr. no.	TMCH: Scanner 1: “GE Medical Senograph System”				TMCH: Scanner 2: “Hologic Selenia System”			
	ROI size	Total number of curvelet coefficients from subbands	Total number of selected curvelet coefficients from subbands	% reduction in curvelet coefficients	ROI size	Total number of curvelet coefficients from subbands	Total number of selected curvelet coefficients from subbands	% reduction in curvelet coefficients
1	459 × 412	11,40,617	7,94,885	30.31	291 × 278	4,89,243	3,17,014	35.20
2	548 × 513	16,93,403	11,17,873	33.99	545 × 246	8,09,627	5,31,604	34.34
3	415 × 303	7,58,323	4,45,585	41.24	560 × 483	16,30,583	10,68,439	34.47
4	645 × 495	19,51,443	11,45,580	41.29	782 × 510	24,00,137	12,75,073	46.87
5	437 × 691	18,16,651	9,85,120	45.77	87 × 141	75,773	67,871	10.43
6	812 × 500	24,39,937	12,87,065	47.25	311 × 185	3,48,565	2,40,546	30.99
7	468 × 379	10,66,333	7,10,242	33.40	262 × 348	5,50,303	2,82,821	48.61
8	673 × 582	23,55,589	17,30,915	26.52	610 × 440	16,14,515	8,42,876	47.79
9	250 × 201	3,04,513	2,35,670	22.61	949 × 391	22,27,209	13,59,338	38.97
10	525 × 488	15,44,691	11,61,942	24.78	365 × 385	8,46,523	6,04,473	28.59
11	488 × 779	22,87,547	16,11,385	29.56	393 × 247	5,85,063	4,90,474	16.17
12	1434 × 966	83,26,581	37,42,277	55.06	341 × 301	6,18,111	4,10,542	33.58
13	348 × 421	8,81,701	6,16,501	30.08	523 × 702	22,06,097	8,00,057	63.73
14	460 × 530	14,67,227	7,99,885	45.48	370 × 284	6,32,539	4,23,727	33.01
15	398 × 450	10,78,441	8,28,064	23.22	344 × 202	4,18,955	3,02,427	27.81
16	247 × 272	4,04,401	3,44,822	14.73	264 × 188	2,99,997	2,31,926	22.69
17	411 × 305	7,57,657	4,05,919	46.42	233 × 247	3,46,983	2,67,686	22.85
18	286 × 344	5,93,155	4,48,566	24.38	370 × 291	6,50,701	4,86,125	25.29
19	417 × 207	5,23,477	4,29,755	17.90	680 × 483	19,79,543	10,52,793	46.82
20	463 × 458	12,75,021	8,57,608	32.74	202 × 266	3,24,295	2,15,042	33.69
Average		16,33,335	9,84,983	33	Average	9,52,738	5,63,543	34

**Table 4:** Number of ROIs resulted in FP reduction using curvelet-based LBP (without sparse) & ANN classification at training and validation stage.

Class	Dataset used	Benign/malignant mass			Nonmass/benign mass			Total (#) images	TPR (true-positive rate) = TP/#lesions	FPPI (false-positive per image) = FP/# images
		Previous stage	Selected (TP)	Lost (FN)	Previous stage	Selected (TN)	Lost (FP)			
Normal vs abnormal	MIAS	108 ∗ 2 = 216	203	13	273	257	16	315	(203/216) = 0.94	(16/315) = 0.05
	DDSM	140 ∗ 4 = 560	465	95	1203	1095	108	240	(465/560) = 0.83	(108/240) = 0.45

Benign vs malignant	MIAS	49	49	0	59	57	2	108	(49/49) = 1.00	(2/108) = 0.02
	DDSM	46 ∗ 2 = 92	91	1	94	91	3	140	(91/92) = 0.99	(3/140) = 0.02

Normal vs malignant	MIAS	49 ∗ 4 = 196	184	12	273	254	19	256	(184/196) = 0.94	(19/256) = 0.07
	DDSM	46 ∗ 4 = 184	180	4	1203	1143	60	146	(180/184) = 0.98	(60/146) = 0.41
	TMCH: Scanner1	107 ∗ 4 = 428	416	12	605	551	54	217	(416/428) = 0.97	(54/217) = 0.25
	TMCH: Scanner2	65 ∗ 4 = 260	255	5	232	214	18	135	(255/260) = 0.98	(18/135) = 0.13

^∗^Augmentation of image.

**Table 5:** Number of ROIs resulted in FP reduction using sparse curvelet coefficient-based LBP & ANN classification at training and validation stage.

Class	Dataset used	Benign/malignant mass			Nonmass/benign mass			Total (#) images	TPR (true-positive rate) = TP/#lesions	FPPI (false-positive per image) = FP/# images
		Previous stage	Selected (TP)	Lost (FN)	Previous stage	Selected (TN)	Lost (FP)			
Normal vs abnormal	MIAS	108 ∗ 2 = 216	201	15	273	265	8	315	(201/216) = 0.93	(8/315) = 0.02
	DDSM	140 ∗ 4 = 560	516	44	1203	1155	48	240	(516/560) = 0.92	(48/240) = 0.2

Benign vs malignant	MIAS	49	48	1	59	59	1	108	(48/49) = 0.98	(1/108) = 0.01
	DDSM	46 ∗ 2 = 92	89	3	94	89	5	140	(89/92) = 0.97	(5/140) = 0.03
Normal vs malignant	MIAS	49 ∗ 4 = 196	192	4	273	259	14	256	(192/196) = 0.98	(14/256) = 0.05
	DDSM	46 ∗ 4 = 184	182	2	1203	1167	36	146	(182/184) = 0.99	(36/146) = 0.25
	TMCH: Scanner1	107 ∗ 4 = 428	424	4	605	593	12	217	(424/428) = 0.99	(12/217) = 0.05
	TMCH: Scanner2	65 ∗ 4 = 260	260	0	232	232	0	135	(260/260) = 1.00	(0/135) = 0

^∗^Augmentation of image.

**Table 6:** Performance evaluation of curvelet-based LBP descriptor algorithm.

Dataset	Classification	Normal-malignant			Normal-abnormal			Benign-malignant		
	Classifier	Sensitivity	Specificity	AUC	Sensitivity	Specificity	AUC	Sensitivity	Specificity	AUC
MIAS	ANN	0.94	0.93	0.94	0.94	0.94	0.94	1.00	0.97	0.99
	SVM	0.85	0.85	0.85	0.83	0.86	0.85	0.88	0.84	0.86
	KNN	0.67	0.63	0.65	0.58	0.57	0.58	0.62	0.68	0.63

DDSM	ANN	0.98	0.95	0.95	0.83	0.91	0.85	0.99	0.97	0.98
	SVM	0.97	0.88	0.92	0.71	0.91	0.83	0.94	0.89	0.92
	KNN	0.96	0.64	0.87	0.67	0.90	0.80	0.87	0.73	0.79

TMCH: Scanner1	ANN	0.97	0.91	0.94	—	—	—	—	—	—
	SVM	0.96	0.91	0.94	—	—	—	—	—	—
	KNN	0.98	0.82	0.89	—	—	—	—	—	—

TMCH: Scanner2	ANN	0.98	0.92	0.96	—	—	—	—	—	—
	SVM	0.97	0.90	0.94	—	—	—	—	—	—
	KNN	0.92	0.83	0.88	—	—	—	—	—	—

**Table 7:** Performance evaluation of proposed algorithm.

Dataset	Classification	Normal-malignant			Normal-abnormal			Benign-malignant		
	Classifier	Sensitivity	Specificity	AUC	Sensitivity	Specificity	AUC	Sensitivity	Specificity	AUC
MIAS	ANN	0.98	0.95	0.96	0.93	0.97	0.95	0.97	1.00	0.98
	SVM	0.88	0.83	0.85	0.85	0.82	0.84	0.84	0.92	0.87
	KNN	0.55	0.51	0.53	0.55	0.63	0.56	0.61	0.67	0.61

DDSM	ANN	0.99	0.97	0.98	0.92	0.96	0.93	0.97	0.95	0.96
	SVM	0.99	0.92	0.96	0.89	0.96	0.92	0.94	0.92	0.93
	KNN	0.98	0.73	0.92	0.74	0.90	0.82	0.89	0.77	0.83

TMCH: Scanner1	ANN	0.99	0.98	0.98	—	—	—	—	—	—
	SVM	0.98	0.96	0.97	—	—	—	—	—	—
	KNN	0.99	0.92	0.95	—	—	—	—	—	—

TMCH: Scanner2	ANN	1.00	1.00	1.00	—	—	—	—	—	—
	SVM	1.00	0.98	0.99	—	—	—	—	—	—
	KNN	0.96	0.92	0.94	—	—	—	—	—	—

**Table 8:** Comparison of classification accuracy, AUC, and FP/image values from different approaches in breast cancer diagnosis.

Author	Database	Method	Classifier	Result	AUC	FP/image
Eltoukhy et al. [33]	MIAS	Biggest curvelet coefficients as a feature vector	Euclidean classifier	94.07%	—	—
Eltoukhy et al. [42]				98.59	—	—
Eltoukhy et al. [8]			SVM	97.3	—	—
Dhahbi et al. [34]	Mini-MIAS	Curvelet moments	KNN	91.27	—	—
	DDSM			86.46	—	—
Bruno et al. [4]	DDSM	Curvelet + LBP	SVM	85	0.85	—
			PL	94	0.94	—
da Rocha et al. [40]	DDSM	LBP	SVM	88.31	0.88	—
Kanadam and Chereddy [3]	MIAS	Sparse ROI	SVM	97.42	—	—
Pereira et al. [18]	DDSM	Wavelet and Wiener filter	Multiple thresholding, wavelet, and GA	—	—	1.37
Liu and Zeng [29]	DDSM, FFDM	GLCM, CLBP, and geometric features	SVM	—	—	1.48
De Sampaio et al. [39]	DDSM	LBP	DBSCAN	98.26		0.19
Zyout et al. [30]	DDSM	Second order statistics of wavelet coefficients (SOSWC)	SVM	96.8	0.97	0.018
	MIAS			95.2	96.6	0.029
Casti et al. [31]	DDSM	Differential features	Fisher linear discriminant analysis (FLDA)	—	—	1.68
	MIAS					2.12
	FFDM					0.82
Proposed method	MIAS	LBP based on sparse curvelet subband coefficients	ANN	98.57	0.98	0.01
	DDSM			98.70	0.98	0.03
	TMCH: Scanner1			98.30	0.98	0.05
	TMCH: Scanner2			100	1	0

## Data Availability

In this research, we have used two publicly available datasets MIAS and DDSM. These datasets can be found here in [17] and [19]. The third database is collected from the local hospital Tata Memorial Cancer Hospital, Mumbai, which can be found at http://eureka.sveri.ac.in/ or available from the corresponding author upon request.

## References

[1] Malvia S., Bagadi S. A., Dubey U. S., Saxena S. (2017). Epidemiology of breast cancer in Indian women. *Asia-Pacific Journal of Clinical Oncology*.

[2] Gupta A., Shridhar K., Dhillon P. (2015). A review of breast cancer awareness among women in India: cancer literate or awareness deficit?. *European Journal of Cancer*.

[3] Kanadam K. P., Chereddy S. R. (2016). Mammogram classification using sparse-ROI: a novel representation to arbitrary shaped masses. *Expert Systems with Applications*.

[4] Bruno D. O. T., do Nascimento M. Z., Ramos R. P. (2016). LBP operators on curvelet coefficients as an algorithm to describe texture in breast cancer tissues. *Expert Systems with Applications*.

[5] Hussain M. (2014). False-positive reduction in mammography using multiscale spatial Weber law descriptor and support vector machines. *Neural Computing and Applications*.

[6] Pawar M. M., Talbar S. N. (2016). Genetic fuzzy system (GFS) based wavelet co-occurrence feature selection in mammogram classification for breast cancer diagnosis. *Perspectives in Science*.

[7] Muramatsu C., Hara T., Endo T., Fujita H. (2016). Breast mass classification on mammograms using radial local ternary patterns. *Computers in Biology and Medicine*.

[8] Eltoukhy M. M., Faye I., Samir B. B. (2012). A statistical based feature extraction method for breast cancer diagnosis in digital mammogram using multiresolution representation. *Computers in Biology and Medicine*.

[9] Li Y., Chen H., Yang Y., Cheng L., Cao L. (2015). A bilateral analysis scheme for false positive reduction in mammogram mass detection. *Computers in Biology and Medicine*.

[10] Gandhamal A., Talbar S., Gajre S., Hani A. F. M., Kumar D. (2017). Local gray level S-curve transformation—a generalized contrast enhancement technique for medical images. *Computers in Biology and Medicine*.

[11] Anand S., Gayathri S. (2015). Mammogram image enhancement by two-stage adaptive histogram equalization. *Optik-International Journal for Light and Electron Optics*.

[12] Pawar M. M., Talbar S. N. (2018). Local entropy maximization based image fusion for contrast enhancement of mammogram. *Journal of King Saud University-Computer and Information Sciences*.

[13] Ganesan K., Acharya U. R., Chua K. C., Min L. C., Abraham K. T. (2013). Pectoral muscle segmentation: a review. *Computer Methods and Programs in Biomedicine*.

[14] Maitra I. K., Nag S., Bandyopadhyay S. K. (2012). Technique for preprocessing of digital mammogram. *Computer Methods and Programs in Biomedicine*.

[15] Oliver A., Freixenet J., Martí J. (2010). A review of automatic mass detection and segmentation in mammographic images. *Medical Image Analysis*.

[16] Görgel P., Sertbas A., Ucan O. N. (2013). Mammographical mass detection and classification using local seed region growing–spherical wavelet transform (lsrg–swt) hybrid scheme. *Computers in Biology and Medicine*.

[17] Suckling J., Parker J., Dance D. (1994). *The Mammographic Image Analysis Society Digital Mammogram Database, International Congress Series*.

[18] Pereira D. C., Ramos R. P., do Nascimento M. Z. (2014). Segmentation and detection of breast cancer in mammograms combining wavelet analysis and genetic algorithm. *Computer Methods and Programs in Biomedicine*.

[19] Heath M., Bowyer K., Kopans D., Moore R., Kegelmeyer P., Yaffe M. J. The digital database for screening mammography.

[20] Rouhi R., Jafari M., Kasaei S., Keshavarzian P. (2015). Benign and malignant breast tumors classification based on region growing and CNN segmentation. *Expert Systems with Applications*.

[21] Berber T., Alpkocak A., Balci P., Dicle O. (2013). Breast mass contour segmentation algorithm in digital mammograms. *Computer Methods and Programs in Biomedicine*.

[22] Rouhi R., Jafari M. (2016). Classification of benign and malignant breast tumors based on hybrid level set segmentation. *Expert Systems with Applications*.

[23] Salazar-Licea L. A., Pedraza-Ortega J. C., Pastrana-Palma A., Aceves-Fernandez M. A. (2017). Location of mammograms ROI’s and reduction of false-positive. *Computer Methods and Programs in Biomedicine*.

[24] Kohonen T. (1998). The self-organizing map. *Neurocomputing*.

[25] Demirhan A., Güler İ. (2011). Combining stationary wavelet transform and self-organizing maps for brain MR image segmentation. *Engineering Applications of Artificial Intelligence*.

[26] Lladó X., Oliver A., Freixenet J., Martí R., Martí J. (2009). A textural approach for mass false positive reduction in mammography. *Computerized Medical Imaging and Graphics*.

[27] Junior G. B., da Rocha S. V., Gattass M., Silva A. C., de Paiva A. C. (2013). A mass classification using spatial diversity approaches in mammography images for false positive reduction. *Expert Systems with Applications*.

[28] Vállez N., Bueno G., Déniz O. (2014). Breast density classification to reduce false positives in CADe systems. *Computer Methods and Programs in Biomedicine*.

[29] Liu X., Zeng Z. (2015). A new automatic mass detection method for breast cancer with false positive reduction. *Neurocomputing*.

[30] Zyout I., Czajkowska J., Grzegorzek M. (2015). Multi-scale textural feature extraction and particle swarm optimization based model selection for false positive reduction in mammography. *Computerized Medical Imaging and Graphics*.

[31] Casti P., Mencattini A., Salmeri M. (2016). Contour-independent detection and classification of mammographic lesions. *Biomedical Signal Processing and Control*.

[32] Beura S., Majhi B., Dash R. (2015). Mammogram classification using two dimensional discrete wavelet transform and gray-level co-occurrence matrix for detection of breast cancer. *Neurocomputing*.

[33] Eltoukhy M. M., Faye I., Samir B. B. (2010). A comparison of wavelet and curvelet for breast cancer diagnosis in digital mammogram. *Computers in Biology and Medicine*.

[34] Dhahbi S., Barhoumi W., Zagrouba E. (2015). Breast cancer diagnosis in digitized mammograms using curvelet moments. *Computers in Biology and Medicine*.

[35] Raghavendra U., Acharya U. R., Fujita H., Gudigar A., Tan J. H., Chokkadi S. (2016). Application of Gabor wavelet and locality sensitive discriminant analysis for automated identification of breast cancer using digitized mammogram images. *Applied Soft Computing*.

[36] Ganesan K., Acharya U. R., Chua C. K., Min L. C., Abraham K. T., Ng K.-H. (2013). Computer-aided breast cancer detection using mammograms: a review. *IEEE Reviews in Biomedical Engineering*.

[37] Abdel-Nasser M., Rashwan H. A., Puig D., Moreno A. (2015). Analysis of tissue abnormality and breast density in mammographic images using a uniform local directional pattern. *Expert Systems with Applications*.

[38] Wajid S. K., Hussain A. (2015). Local energy-based shape histogram feature extraction technique for breast cancer diagnosis. *Expert Systems with Applications*.

[39] de Sampaio W. B., Silva A. C., de Paiva A. C., Gattass M. (2015). Detection of masses in mammograms with adaption to breast density using genetic algorithm, phylogenetic trees, LBP and SVM. *Expert Systems with Applications*.

[40] da Rocha S. V., Junior G. B., Silva A. C., de Paiva A. C., Gattass M. (2016). Texture analysis of masses malignant in mammograms images using a combined approach of diversity index and local binary patterns distribution. *Expert Systems with Applications*.

[41] Ojala T., Pietikäinen M., Harwood D. (1996). A comparative study of texture measures with classification based on featured distributions. *Pattern Recognition*.

[42] Eltoukhy M. M., Faye I., Samir B. B. (2010). Breast cancer diagnosis in digital mammogram using multiscale curvelet transform. *Computerized Medical Imaging and Graphics*.

[43] Ojala T., Pietikainen M., Maenpaa T. (2002). Multiresolution gray-scale and rotation invariant texture classification with local binary patterns. *IEEE Transactions on Pattern Analysis and Machine Intelligence*.

[44] Candès E., Demanet L., Donoho D., Ying L. (2006). Fast discrete curvelet transforms. *Multiscale Modeling & Simulation*.

[45] Dudhane A., Shingadkar G., Sanghavi P., Jankharia B., Talbar S. Interstitial lung disease classification using feed forward neural networks.

[46] Dudhane A. A., Talbar S. N. Multi-scale directional mask pattern for medical image classification and retrieval.

[47] http://eureka.sveri.ac.in/

